# Healthy Dietary Patterns and Oxidative Stress as Measured by Fluorescent Oxidation Products in Nurses’ Health Study

**DOI:** 10.3390/nu8090587

**Published:** 2016-09-21

**Authors:** Seungyoun Jung, Stephanie A. Smith-Warner, Walter C. Willett, Molin Wang, Tianying Wu, Majken Jensen, Susan E. Hankinson, A. Heather Eliassen

**Affiliations:** 1 Departments of Nutrition, Harvard T.H. Chan School of Public Health, Boston, MA 02115, USA; swarner@hsph.harvard.edu (S.A.S.-W.); wwillett@hsph.harvard.edu (W.C.W.); 2 Departments of Epidemiology, Harvard T.H. Chan School of Public Health, Boston, MA 02115, USA; stmow@channing.harvard.edu (M.W.); majken.jensen@channing.harvard.edu (M.J.); nhahe@channing.harvard.edu (A.H.E.); 3 Department of Epidemiology and Public Health, University of Maryland Baltimore School of Medicine, Baltimore, MD 21201, USA; 4 Channing Division of Network Medicine, Department of Medicine, Brigham and Women’s Hospital, Boston, MA 02115, USA; sue.hankinson@channing.harvard.edu; 5 Department of Environmental Health, University of Cincinnati Medical Center, Cincinnati, OH 45219, USA; wutg@ucmail.uc.edu; 6 Division of Biostatistics and Epidemiology, University of Massachusetts, Amherst, MA 01003, USA

**Keywords:** healthy eating pattern, oxidative stress, fluorescent oxidation products, nutrition, epidemiology

## Abstract

Healthy diets may lower oxidative stress and risk of chronic diseases. However, no previous studies examined associations between diet and fluorescent oxidation products (FlOP), a global marker of oxidative stress. We evaluated associations between healthy eating patterns (Alternative Healthy Eating Index (AHEI), Dietary Approach to Stop Hypertension (DASH), and Alternate Mediterranean Diet (aMED)) and FlOP, measured at three excitation/emission wavelengths (FlOP_360, FlOP_320, FlOP_400) from 2021 blood samples collected from 1688 women within the Nurses’ Health Study. AHEI, DASH, and aMED scores were significantly positively associated with FlOP_360 and FlOP_320 concentrations (*p*-trend ≤ 0.04), but not associated with FlOP_400. Among specific food groups that contribute to these diet scores, significantly positive associations were observed with legumes and vegetables for FlOP_360, vegetables and fruits for FlOP_320, and legumes and alcohol for FlOP_400. Inverse associations were observed with nuts, sweets or desserts, and olive oil for FlOP_360, nuts for FlOP_320 and sweets or desserts for FlOP_400 (all *p*-trend ≤ 0.05). However, FlOP variation due to diet was small compared to overall FlOP variation. In conclusion, AHEI, DASH, and aMED scores were unexpectedly positively, but weakly, associated with FlOP_360 and FlOP_320. However, these findings should be interpreted cautiously as the determinants of FlOP concentrations are not fully understood.

## 1. Introduction

Oxidative stress is a condition associated with an increased rate of cellular damage induced from an imbalance between the overproduction of reactive oxygen species and insufficient removal of oxidants by defense systems [1]. Oxidative stress damages lipid, protein, and DNA integrity [2,3], which subsequently disrupt inflammation, tissue homeostasis, apoptosis, and proliferation [4,5] and has been implicated in the pathophysiology of a myriad of human diseases [1,2,5,6]. Foods rich in healthy diets—characterized by a high intake of fruit, vegetables, and whole grains as well as a low intake of saturated fat—are aggregate sources of bioactive compounds. These compounds work as components in antioxidant systems, such as superoxide dismutases or glutathione peroxidase, and may break free radical chain reactions [7]. Therefore, the reduced risk of chronic diseases such as cancer, cardiovascular disease and neurodegenerative disorders observed with healthy diet [8,9,10,11,12,13] have been postulated to be, in part, attributable to their effect on lowering oxidative stress. However, epidemiologic evidence supporting the beneficial effects of healthy diets on oxidative stress was primarily from small and short-term feeding trials [14,15,16,17,18,19,20,21,22].

Fluorescent oxidation products (FlOP) are a global marker of oxidative stress. Compared to commonly used oxidative stress biomarkers that reflect oxidative damage to specific cellular components, experimental studies demonstrated that reactive oxygen species, such as aldehydes or hydroperoxides, interact with subcellular components—including amino groups, fatty acids and DNA—to increase the formation of fluorescent products [23,24,25,26]. Thus, higher FlOP concentrations reflect higher levels of oxidative stress that arises from multiple cellular components. Epidemiologic studies also reported that FlOPs are significantly positively associated with diseases or conditions related to oxidative stress, such as smoking [27], hypertension [27], coronary heart disease [28,29] and Alzheimer’s disease [30]. Furthermore, FlOPs were observed to be reasonably reproducible [29] and relatively stable regardless of processing time and collection matrix [31]. Therefore, FlOPs are biologically sensitive and reliable biomarkers of global oxidative stress.

To our knowledge, no epidemiologic studies have examined the association between diet and FlOP concentrations in healthy women, though in vitro and in vivo studies reported that antioxidants high in healthy diets—such as β-carotene and vitamin E—reduce oxidative stress measured by FlOP [24,32]. Examining the association between FlOP concentrations and dietary patterns in a large sample of healthy women can improve our understanding of the mechanisms underlying the benefit of a healthy diet. Therefore, we hypothesized that healthy dietary patterns are inversely associated with FlOP. To evaluate our hypothesis, we conducted cross-sectional analyses examining the association between FlOP concentrations and the established healthy dietary patterns associated with the lower risk of chronic diseases [8,9,10,11,12] (the Alternative Healthy Eating Index (AHEI), the Dietary Approach to Stop Hypertension (DASH), and the Alternate Mediterranean Diet (aMED)) using a total of 2021 blood samples collected from 1688 healthy women who were free of disease at the time of blood collection in the Nurses’ Health Study. We further examined the association between individual food groups that form these diet patterns and plasma FlOP.

## 2. Materials and Methods

### 2.1. The Nurses’ Health Study Blood Subcohort

The Nurses’ Health Study (NHS) enrolled 121,700 female nurses aged from 30 to 55 years in 1976. Participants provided information on lifestyles and medical histories at baseline and subsequent biennial questionnaires [33]. Blood samples were first collected from 32,826 women from 1989 to 1990 [34,35]. Among those, 18,743 women provided second blood samples from 2000 to 2002 [36]. All samples were obtained and processed using identical protocol in both blood collections and samples packed on ice were mailed to the laboratory via overnight couriers. Ninety-seven percent of them arrived within 26 hours of being drawn. Upon arrival, samples were processed, separated into plasma—red blood cell and white blood cell components—and archived in liquid nitrogen freezers (≤−130 °C).

### 2.2. Study Population

This study included women within nested case-control studies of fluorescent oxidation products (FlOP) and myocardial infarction (MI) [29] and breast cancer in the NHS [37]. We included all controls and cases of MI and breast cancer diagnosed at least 10 years after the blood collection (*n* = 2144). FlOP concentrations in the MI nested case-control studies were assayed in the 1990 blood samples (*n* = 1019), whereas FlOP concentrations in the breast cancer nested case-control studies were measured in both the 1990 and 2000 blood samples (*n* = 1125). Among those, controls who were subsequently diagnosed with MI or breast cancer (*n* = 13) were excluded. Outliers for FlOP_360, FlOP_320, and FlOP_400—identified by the extreme studentized deviate many-outlier approach [38]—were set to missing (*n* = 14 for FlOP_360, 11 for FlOP_320, and 14 for FlOP_400), leaving a total of 2031 samples with any FlOP measurements. We further excluded women who reported an implausible intake of total calories (<800 or >4200 kcal/day) or missed more than 70 items on the food frequency questionnaire (FFQ) (*n* = 105) and who had a history of MI or cancer before the blood collection (*n* = 5). This left a total of 2021 blood samples collected from 1688 women who were free of cancer and MI at the time of data collection (Figure S1). The institutional review board of the Brigham and Women’s Hospital (Boston, MA, USA) approved this study.

### 2.3. Assessment of Dietary Intake and Healthy Eating Diet Score

Usual dietary intake was assessed by a validated FFQ, administered approximately every four years in the NHS [39]. This present study used dietary intakes assessed from 1990 FFQ and 2000 FFQ. Nutrient intake was calculated by multiplying the frequency of consumption of each specified food item by the nutrient content of the specified portions and summing these products for all food items. Intake of the food group was calculated by summing the consumption of all individual food items within the specific group. The multivitamins and other dietary supplements were assessed via biennial questionnaires. Nutrient intake was energy-adjusted [40]. The correlation coefficients of the nutrient intakes assessed from the FFQ and dietary records ranged from 0.36 to 0.75 [39].

AHEI, aMED and DASH scores (Table S1) were calculated using the FFQ assessment collected closest to the time of blood collection. AHEI scores are based on nine components [41,42]: vegetables (excluding potatoes), fruits, nuts and soy, white to red meat ratio, trans fat, polyunsaturated to saturated fat ratio, cereal fiber, alcohol, and multivitamin use. For each item, a score from 1 to 10 was assigned, with 10 indicating the most desirable consumption (e.g., high vegetable intake or low trans fat intake). For multivitamin use, the possible scores were 7.5 for a multivitamin user of more than five years and 2.5 for others.

The aMED score is composed of nine items [42,43]: vegetables (excluding potatoes), fruits, nuts, legumes, whole grains, monounsaturated to saturated fat ratio, fish, red or processed meats, and alcohol. For red or processed meats, one point was given when intake was less than the median intake. For alcohol, one point was assigned for intakes within the ideal range of intake (5–15 g/day). For the remaining items, one point was given for each desirable component if the participant’s intake of that item was greater than the median; otherwise, no point was assigned.

The DASH score [44,45] is composed of eight items: vegetables (excluding potatoes), fruits, nuts or legumes, whole grains, low-fat dairy products, red or processed meats, sodium, and sugar-sweetened beverages. A score from one to five was assigned, with five indicating the most desirable consumption. For vegetables, fruits, nuts or legumes, whole grains, and low-fat dairy products, a value of one was assigned for the lowest quintile intake of that food. For red or processed meats, sodium, and sugar-sweetened beverages, inverse scores were assigned.

### 2.4. Laboratory Assays

FlOP was measured using the assay methods, proven to reflect the change of oxidative insults [31], in Dr. Wu’s laboratory. Plasma samples extracted with ethanol-ether were measured by a spectrofluorometer at the wavelengths (excitation/emission) of 360/420 nm (FlOP_360), 320/420 nm (FlOP_320), and 400/475 nm (FlOP_400). The fluorescence was determined as relative fluorescent intensity (FI) units per milliliter of plasma. FlOP_360 reflects the oxidation product arising from the interaction of lipid oxidative products with protein, DNA, and carbohydrates [27,28,31]. FlOP_320 represents the interaction of lipid hydroperoxide with DNA and metals [24,25]. FlOP_400 represents the interaction of malondialdehyde (MDA) with proteins and phospholipids [25,46]. The correlation coefficients with FlOP_360 were 0.44 for FlOP_320 and 0.76 for FlOP_400, whereas the correlation between FlOP_320 and FlOP_400 was 0.17. The average of intra-batch coefficients of variation (CV) in the MI and breast cancer nested case-control studies ranged from 5.7% to 13.6%. To correct for variation in FlOP concentrations across batches, we standardized FlOP concentrations to an average batch [47].

Plasma total cholesterol and carotenoids were measured in a subset of the study population. Cholesterol was measured by enzymatic determination using reagents from Roche Diagnostics (Indianapolis, IN, USA) and Genzyme Corporation (Cambridge, MA, USA) [48]. CVs, based on blinded quality control samples, were 2%–14%. Plasma carotenoids were assayed using reverse-phase HPLC [49]. CVs were generally <20% with a maximum CV of 41% for β-cryptoxanthin.

### 2.5. Assessment of Non-Dietary Data

Lifestyle factors, such as physical activity and smoking status, were reported at baseline and updated via biennial follow-up questionnaires. Information on age, fasting status, and time of day at blood draw were collected through separate questionnaires. Body mass index (BMI) was calculated using the weight reported at blood draw and height at baseline.

### 2.6. Statistical Analyses

FlOP concentrations were log-transformed to improve normality. To estimate the association of healthy dietary scores with FlOP concentrations, we used dietary intake and covariate information collected closest to the time of blood draw. For example, samples collected in 1990 were matched to dietary intake or covariate data obtained from the 1988 or 1990 questionnaires, whereas samples collected in 2000 were matched to corresponding data from the 1998 or 2000 questionnaires. The geometric means of FlOP concentrations were calculated using a linear mixed model with a sandwich variance estimator [50]. The linear mixed model included a random intercept to take into account the correlation among the repeated measurements. Multivariate models adjusted for variables listed in Table 2 as fixed effects. The trend test was conducted using quintile medians, modeled as continuous variables [51]. We also conducted an analysis stratified by multivitamin use (yes/no), smoking status (never/ever), alcohol consumption (none/>0–<10/≥10 g/day), BMI (<25/≥25 kg/m^2^), blood collection year (1990/2000), and age (<60/≥60 years). The interactions between dietary pattern scores and these stratification factors were tested by including the cross-product term between these factors and the dietary pattern score in the multivariable model. In sensitivity analyses, we restricted our analysis to controls or women who fasted at the time of blood collection and repeated the analyses.

All analyses were conducted separately for FlOP_360, FlOP_320, and FlOP_400. However, results from FlOP_360 are presented as our primary analysis given that FlOP_360 is the most comprehensive biomarker of oxidative products [27,28,31] validated previously [28]; FlOP_320 and FlOP_400 were analyzed as secondary outcomes. All tests were two-sided and considered to be statistically significant if *p* < 0.05. Analyses were performed using SAS 9.2 (SAS Institute Inc., Cary, NC, USA).

## 3. Results

Women in the higher quintiles of healthy eating scores were less likely to be current smokers or have high-pack years of smoking. They were also more likely to be physically active, use multivitamins, and drink alcohol; but among alcohol consumers, women in the highest quintiles of healthy eating scores drank less alcohol than those in the lowest quintiles (Table 1). The BMI, fasting status, and age at blood draw were similar across quintiles of dietary scores. The majority of women (~90%) were not using medications such as cholesterol-lowering drugs or anti-hypertensive drugs (data not shown).

Results from the multivariate and simple models adjusted for age and blood collection variables were generally similar (data not shown). We, therefore, present only multivariate results. AHEI, DASH, and aMED scores were positively associated with FlOP_360 (*p*-trend ≤ 0.03) (Table 2). When comparing women in the extreme quintiles of dietary scores (Q1–Q5), the mean FlOP_360 concentrations increased from 224 to 240 FI/mL for AHEI (mean difference = 16 FI/mL), from 222 to241 FI/mL for DASH (mean difference = 19 FI/mL), and from 224 to 243 FI/mL for aMED (mean difference = 19 FI/mL). Positive associations were observed between the three dietary patterns and FlOP_320 (*p*-trend ≤ 0.04), whereas FlOP_400 was not associated with any of the three dietary patterns. The overall differences in FlOP_360, FlOP_320, and FlOP_400 across extreme quintiles of dietary patterns were consistently small (<19 FI/mL for FlOP_360, <109 FI/mL for FlOP_320 and <2 FI/mL for FlOP_400) compared to the overall variation in FlOP concentrations (the differences between the 90th and 10th percentiles of FlOP: 218 FI/mL for FlOP_360, 2484 FI/mL for FlOP_320, and 66 FI/mL for FlOP_400).

Given that the AHEI and aMED diets award points for moderate alcohol consumption, but alcohol may contribute to oxidative stress; we also ran analyses taking out the alcohol components for AHEI and aMED. However, we obtained similar results (data not shown). Additional adjustments for multivitamin use, history of hypertension and diabetes, and plasma cholesterol and carotenoids also did not change results materially (data not shown). Restricting the analysis to fasting samples [27] attenuated the associations between healthy eating scores and FlOP_360 and FlOP_320 measurements, but the associations remained in the same positive direction (data not shown). Other analyses excluding cases or users of anti-hypertensive or cholesterol lowering medications did not change results (data not shown). Multivitamin use, smoking status, alcohol consumption, BMI, blood collection year, and age at blood draw did not significantly modify associations between healthy dietary patterns and FlOP concentrations, except for significant interactions of AHEI score with smoking status in relation to FlOP_400 and of the DASH score with BMI in relation to FlOP_320 (*P-*interaction ≤ 0.01; Table S2). However, the associations between the AHEI score and FlOP_400 were non-significant in each stratum of smoking status. In the stratified analyses of BMI, the positive association between the DASH score and FlOP_320 was only statistically significant in women with BMI ≥ 25 kg/m^2^ (Table S2).

To identify foods that contributed to the observed association between the dietary patterns and FlOP concentrations, we examined the associations between the food groups that contributed to the dietary pattern scores and FlOP concentrations (Table 3; Table S3). Intakes of total vegetables and legumes were significantly positively associated with FlOP_360 (*p*-trend < 0.002), whereas intakes of nuts, sweets or desserts, and olive oil were significantly inversely associated with FlOP_360 (*p*-trend < 0.01). No significant associations were observed between FlOP_360 and consumption of total fruits, whole grains, red/processed meat, poultry, fish, alcohol, or sugar-sweetened beverages. The differences in the means of FlOP_360 concentrations across quintiles of individual food groups were less than 27 FI/mL. Intakes of total vegetables, particularly cruciferous (e.g., broccoli, cabbage, kale) and other (e.g., eggplant, celery, mixed vegetable) vegetables, and total fruits were significantly positively associated and nut intake was inversely associated with FlOP_320 (*p*-trend ≤ 0.008). There were generally no associations between individual food groups and FlOP_400 except a significant positive association with alcohol consumption and a significant inverse association with intake of sweets or desserts (*p*-trend ≤ 0.04).

When we examined individual nutrients (Table S4), intakes of saturated fat, trans fat, and protein were significantly inversely with FlOP_360, while dietary fiber was positively associated (*p*-trend ≤ 0.04). Among the antioxidants, vitamin C, lutein/zeaxanthin, and lycopene intakes were significantly positively associated with FlOP_360 (*p*-trend ≤ 0.04). Nonetheless, the differences in the geometric means of FlOP_360 across quintiles of nutrients were less than 28 FI/mL. Similarly, FlOP_320 was significantly positively associated with protein, dietary fiber, vitamin C and lutein/zeaxanthin. Vitamin E, vitamin A, β-carotene, and total carotenoid intakes were more strongly associated with FlOP_320 than FlOP_360 (*p*-trend ≤ 0.03). No significant association was observed for FlOP_400 with any of the nutrients examined, except for an inverse association with flavonoids (*p*-trend = 0.006). However, these nutrients contributed to only small variations in FlOP concentrations (model *r*^2^ ≤ 0.03 for FlOP_360 and FlOP_320 and ≤0.008 for FlOP_400; data not shown).

## 4. Discussion

In this large, cross-sectional study of healthy women, we examined the association between healthy eating patterns and oxidative stress measured by plasma FlOP concentrations. The AHEI, DASH, and aMED scores were significantly positively associated with FlOP_360 and FlOP_320. Higher intake of total vegetables and legumes and lower intakes of nuts and sweets or desserts were significantly associated with higher FlOP_360 concentrations. Lower intakes of nuts and higher intakes of total vegetables (particularly cruciferous and other vegetables) and total fruits were significantly associated with higher FlOP_320. Among the nutrients examined, positive associations were observed for the intakes of protein, dietary fiber, vitamin C and lutein/zeaxanthin with FlOP_360 and FlOP_320. The three dietary scores, individual foods, and nutrients were generally not associated with FlOP_400. Nonetheless, the variation of FlOP_360, FlOP_320 and FlOP_400 explained by these dietary predictors was small and sometimes opposite to the expected direction, suggesting that FlOP may not be a useful measure of diet-related oxidative stress in population studies.

To date, a variety of oxidative stress biomarkers have been used, though each reflects different sources of oxidative stress. For example, FlOP represents the end product of reactions between the free radicals and biologic molecules from the multiple cellular components of lipid, protein, and DNA, whereas malondialdehyde (MDA) measures an oxidative product from all polyunsaturated fat. F2-iso-prostanes represents oxidation of arachidonic acid and 8OHdG excretion reflects oxidative damage to DNA [52]. Furthermore, the behavior of oxidative biomarkers can differ by population characteristics such as age [53], and a particular exposure may variably affect markers of oxidative stress [54].

Eight small intervention studies examined the effect of the Mediterranean diet on MDA [17,55], F2-isoprostanes [18,56], 8OHdG [14,15], or oxidized LDL [16,19]. Among those, six studies [14,15,16,17,18,19] observed reduced levels of oxidative stress in the Mediterranean diet group, compared to the control diet group. In the remaining two studies, the level of oxidative stress did not differ between the intervention and control arms [55,56]. The recent randomized controlled trial of 192 obese participants (with two years of follow-up) found that levels of F2-isoprostanes significantly decrease in the intervention group with the Mediterranean diet, compared to the control group with a conventional diet [18]. All three previous DASH diet intervention studies [20,21,22] found a decreased level of either MDA or F2-isoprostanes when comparing the intervention group to the control group. Furthermore, previous intervention studies of high fruit and vegetable consumption generally reported a favorable effect on oxidative stress as evidenced by either lower oxidized LDL or increased antioxidant capacity [57,58,59,60,61,62,63,64,65,66]. In summary, current evidence generally supports the benefit of healthy diet-lowering oxidative stress, contrary to the positive associations observed in our study.

Previously, the potential adverse effects of natural toxins in plants [67,68], pesticide exposure on fruits and vegetables [69], and the high nitrate content of vegetables [70] were reported. In the Alpha-Tocopherol, Beta Carotene Cancer Prevention study [71] and the Beta Carotene and Retinol Efficacy Trial [71], smokers who were administered beta-carotene supplementation were observed to have a higher incidence of lung cancer than smokers administered a placebo, suggesting a detrimental potential of carotene under certain oxidative conditions. However, these study results are not comparable with our findings, given the higher doses achieved from supplements compared to dietary consumption, as well as the fact that all study participants were smokers. Common levels of fruit and vegetable consumption are also not likely to be sufficient to induce toxicity.

Reviewing the evidence from animal and epidemiologic studies, it is unlikely that healthy eating scores increase oxidative stress. Rather, our results suggest that, although diet may lower risk of chronic disease, the involved mechanism may not be oxidative stress as measured by plasma FlOP. FlOP may not serve as a biomarker of oxidative stress for dietary exposure. Indeed, the magnitude of increase in FlOP_360, FlOP_320, and FlOP_400 across extreme quintiles of dietary patterns, individual foods, and nutrients was small, compared to overall variation of FlOP defined by inter-decile range (<28 FI/mL vs. 352 FI/mL for FlOP_360, <213 FI/mL vs. 2483 FI/mL for FlOP_320 and <6 FI/mL vs. 66 FI/mL for FlOP_400). Similarly, the differences in FlOP due to diet are far less than the FlOP differences we observed previously due to smoking, hypertension, and coronary heart disease risk [27,28]. Thus, positive associations observed between diet and the FlOP in our analyses were weak and may not be clinically meaningful despite the statistical significance.

Alternatively, although the FlOP assay is a sensitive, global marker of oxidative stress that captures the final fluorescent oxidation products resulting from several cellular components [72,73], little is known about the specificity of FlOP measurements. Our results might be partly attributable to other fluorescent compounds that are irrelevant to oxidative damage [72,74]. For example, dietary factors such as lycopene, vitamin C, and protein that were selected as predictors of FlOP in our study have delocalized systems of conjugated bonds or aromatic organic compounds that can also emit fluorescence [75,76]. Certain organic radicals reduced by antioxidants may also emit fluorescence. These may interfere with the FlOP assay [74] in their performance measuring the accumulation of oxidation products [31]. Thus, the weak association between dietary factors and FlOP concentrations could have been more easily affected by the possible potential dietary fluorescent compounds or chance findings detected in the FlOP assay, leading to the unexpected results of our study.

Our study has several limitations. Though we cannot rule out some unknown preexisting conditions that might have influenced FlOP levels, this cross-sectional analysis is based on the data and blood specimen collected from healthy women. In addition, we further excluded women who developed MI and breast cancer within 10 years of blood collection to avoid any behavior change due to preclinical disease. The results from control women who did not develop MI or breast cancer also did not change results. One measurement of diet and of the FlOP might have attenuated the association. However, the correlations of individual food intakes from FFQ and from dietary records were generally greater than 0.5 [77]. The intra-class correlation coefficients of FlOP concentrations measured one to two years apart were also reasonable (*r* = 0.44 for FlOP_360; 0.55 for FlOP_320; and 0.70 for FlOP_400) [37]. Although plasma measures of dietary components may differ from the consumption measured by FFQ due to differences in bioavailability [78], neither adjustment for plasma carotenoids (a marker of fruits and vegetables intake) nor for plasma cholesterol (which can influence plasma levels of carotenoids) changed the results. The score ranges, particularly from the aMED and DASH diet, were narrow, limiting the power of our study. However, overall results were similar to those from the AHEI score that has a larger range. Dietary scores pool the intakes of many factors and thus might mask effects of specific foods. However, FlOP concentrations were only weakly associated with the individual food groups and nutrients examined. Finally, though the generalizability of our results could be limited because our study population is health professionals, who are more likely to be well-nourished, the self-reported data from these individuals are known to be highly accurate [79,80] ensuring the validity of the results.

To our knowledge, this is the first and largest epidemiologic study examining the associations of dietary patterns with oxidative stress measured by FlOPs. FlOP is a novel biomarker that is well correlated with established causes of oxidative stress (e.g., smoking, hypertension, reduced renal function) [28]. FlOP also has the clinical and practical benefits of improved sensitivity and stability over other oxidative stress biomarkers [31], but little is known about its dietary determinants. Our analyses contributed to the limited literature on the predictors of FlOP by examining associations between three FlOP measures and dietary pattern scores, food groups, and specific nutrients.

## 5. Conclusions

In conclusion, healthy dietary patterns were not associated with a lower level of oxidative stress measured by FlOPs. We unexpectedly observed the positive associations between AHEI, DASH, and aMED scores and oxidative stress as measured by FlOP_360 and FlOP_320. However, the proportion of FlOP variations determined by dietary factors was low. Our results should be interpreted with caution due to the possibility of potential artifacts or non-specificity of the FlOP biomarkers, and should not alter the current guidelines recommending diets high in fruits, vegetables, whole grains and plant sources of protein.

## Figures and Tables

**Table 1:** Age-standardized characteristics among women * from breast cancer and MI nested case-control analyses in the Nurses’ Health Study according to quintiles (Q) of dietary scores.

	AHEI			DASH			aMED		
	Q1	Q3	Q5	Q1	Q3	Q5	Q1	Q3	Q5
*N*	405	404	404	304	470	492	366	381	566
Age at blood draw, year ^†^	58.2 ± 8.2	59.6 ± 7.4	61.1 ± 7.1	57.7 ± 8.4	59.7 ± 7.4	61.0 ± 6.8	57.6 ± 7.7	60.0 ± 8.0	60.9 ± 7.0
Premenopausal, %	13	14	13	14	16	13	15	14	13
Nonfasting at blood collection, %	27	25	26	27	24	25	28	27	24
Current smoker, %	23	15	10	23	17	12	27	16	9
Pack years of smoking, year	15.4 ± 21.8	14.7 ± 18.9	13.5 ± 18.6	17.4 ± 22.3	13.5 ± 19.3	11.9 ± 17.0	16.1 ± 22.5	15.4 ± 20.4	10.5 ± 16.0
Nondrinker, %	49	33	28	39	36	34	42	35	30
BMI at blood draw, kg/m^2^	26.0 ± 5.1	25.2 ± 4.2	24.9 ± 3.9	26.0 ± 5.1	25.5 ± 4.4	24.8 ± 4.0	25.9 ± 4.7	25.4 ± 4.7	25.0 ± 4.1
Physical activity, MET-h/week ^‡^	13.1 ± 17.2	16.3 ± 19.1	24.1 ± 32.7	10.9 ± 14.0	16.8 ± 19.6	21.7 ± 22.0	13.4 ± 16.9	16.7 ± 20.0	22.2 ± 28.7
Multivitamin usage, %	36	46	47	38	39	48	36	43	47
Energy intake, kcal/d	1713 ± 480	1773 ± 475	1866 ± 522	1748 ± 478	1766 ± 514	1870 ± 478	1555 ± 434	1741 ± 485	2021 ± 489
Alcohol consumption among drinkers, g/day	12.4 ± 14.3	8.0 ± 9.4	7.4 ± 7.1	10.1 ± 12.2	10.2 ± 10.5	8.1 ± 9.6	9.7 ± 12.3	8.9 ± 9.9	8.3 ± 8.1
Vegetables, servings/day	2.3 ± 1.1	3.4 ± 1.7	4.9 ± 2.1	2.2 ± 1.1	3.4 ± 1.6	4.8 ± 1.9	2.1 ± 0.9	3.3 ± 1.6	4.8 ± 1.9
Fruits, servings/day	1.1 ± 0.7	1.7 ± 1.0	2.6 ± 1.3	0.9 ± 0.6	1.6 ± 0.9	2.5 ± 1.2	1.0 ± 0.6	1.6 ± 1.0	2.4 ± 1.2

AHEI, the Alternative Healthy Eating Index diet; aMED, the Alternate Mediterranean Diet; DASH, the Dietary Approach to Stop Hypertension diet; MI, Myocardial Infarction. Values are mean (SD) or percentages and are standardized to the age distribution of the study population. * 2021 FlOP samples from 1688 women were included in this analysis; ^†^ Value is not age adjusted; ^‡^ Metabolic equivalents from recreational and leisure time activities.

**Table 2:** Multivariate-adjusted * geometric mean concentrations of FlOP_360, FlOP_320 and FlOP_400 (FI/mL) by quintiles (Q) of healthy dietary scores from the Nurses’ Health Study.

	Quintiles of Dietary Scores					
	Q1	Q2	Q3	Q4	Q5	*p*-Trend ^†^
AHEI						
*N*	405	403	404	405	404	
Median value of AHEI score	35	43	49	55	64	
FlOP_360 ^‡^ (95% CI)	224 (215, 233)	226 (216, 235)	234 (224, 244)	240 (230, 250)	240 (229, 250)	0.006
FlOP_320 ^‡^ (95% CI)	501 (458, 549)	519 (471, 571)	546 (493, 604)	600 (541, 665)	610 (544, 684)	0.003
FlOP_400 ^‡^ (95% CI)	64 (62, 66)	64 (61, 67)	64 (62, 67)	66 (64, 68)	63 (61, 66)	0.90
DASH						
*N*	304	431	470	324	492	
Median value of DASH score	2	3	4	5	6	
FlOP_360 (95% CI)	222 (212, 233)	232 (224, 241)	231 (223, 240)	230 (220, 242)	241 (231, 251)	0.03
FlOP_320 (95% CI)	515 (462, 574)	528 (482, 579)	545 (496, 598)	559 (499, 627)	607 (549, 672)	0.02
FlOP_400 (95% CI)	64 (61, 66)	66 (63, 68)	65 (63, 68)	62 (60, 64)	64 (62, 66)	0.41
aMED						
*N*	366	327	381	381	566	
Median value of aMED score	20	23	26	28	32	
FlOP_360 (95% CI)	224 (215, 233)	227 (217, 238)	225 (217, 233)	237 (226, 249)	243 (234, 252)	0.002
FlOP_320 (95% CI)	507 (460, 559)	539 (482, 602)	542 (491, 598)	584 (523, 653)	581 (529, 639)	0.04
FlOP_400 (95% CI)	65 (62, 67)	64 (61, 67)	62 (60, 64)	65 (62, 68)	65 (63, 68)	0.54

AHEI, the alternative healthy eating index diet; aMED, the alternate Mediterranean Diet; DASH, the dietary approach to stop hypertension diet; FlOP, fluorescent oxidation product. * Adjusted for age at blood draw (continuous), endpoint and lab project (MI1, MI2, Breast One and Breast Two), fasting status (yes and no), time of blood draw (midnight-11 h, and noon-23 h), season of blood draw (spring, summer, fall, and winter), BMI at blood draw (<20, 20–<25, 25–<30, and ≥30 kg/m^2^), smoking (never, past, and current), physical activity (≤3, 3–<9, 9–<18,18–<27, and ≥27 METs/week), total calorie intake (quintiles). Alcohol consumption (0, >0–<5, 5–<15, 15–<30, and ≥30 g/day) was adjusted for the analyses of the DASH diet score, but not for the analysis of the AHEL and aMED diet scores because alcohol consumption is a component of the AHEL and aMED scores; ^†^ *p*-trend was calculated by modeling the median value of dietary score quintiles as a continuous variable and calculating the Wald test statistic; ^‡^ The mean (SD) of FlOP concentrations was 269 (283) for FlOP_360, 1567 (4287) for FlOP_320, and 72 (54) for FlOP_400.

**Table 3:** Multivariate-adjusted * geometric mean concentrations of FlOP_360 (FI/mL) by quintile (Q) of food group or individual food intake from the Nurses’ Health Study.

Food Group/Individual Food ^‡^	Quintiles of Dietary Intake					*p*-Trend ^†^
	Q1	Q2	Q3	Q4	Q5	
Total vegetables	226 (217, 236)	225 (216, 234)	231 (221, 240)	232 (222, 241)	249 (237, 261)	0.002
Yellow/orange vegetables	234 (222, 246)	229 (221, 238)	232 (223, 242)	235 (225, 247)	232 (224, 241)	0.70
Leafy vegetables	241 (227, 255)	230 (220, 240)	229 (222, 237)	235 (226, 245)	230 (221, 240)	0.76
Cruciferous vegetables	230 (221, 239)	224 (215, 234)	237 (228, 247)	227 (219, 236)	243 (232, 254)	0.08
Other vegetables	230 (220, 241)	230 (221, 239)	230 (220, 240)	228 (221, 236)	243 (231, 255)	0.11
Total fruits	230 (221, 239)	224 (215, 234)	237 (228, 247)	227 (219, 236)	243 (232, 255)	0.07
Nut	245 (233, 256)	232 (223, 241)	231 (221, 241)	234 (225, 243)	222 (213, 231)	0.01
Legume	216 (205, 228)	226 (218, 233)	228 (218, 238)	241 (232, 251)	243 (233, 253)	<0.001
Whole grains	233 (221, 246)	233 (223, 244)	236 (222, 250)	227 (219, 236)	233 (226, 241)	0.65
Red/processed meat	228 (219, 238)	231 (221, 241)	235 (226, 245)	240 (229, 251)	226 (217, 236)	0.72
Poultry	246 (231, 262)	224 (215, 234)	224 (216, 234)	229 (222, 236)	243 (233, 254)	0.15
Fish	227 (216, 239)	236 (226, 246)	226 (219, 234)	240 (230, 250)	230 (221, 240)	0.74
Alcohol	229 (221, 237)	228 (221, 234)	244 (234, 255)	231 (219, 245)	241 (221, 264)	0.07
Sugar-sweetened beverages	228 (219, 238)	231 (221, 241)	235 (226, 245)	240 (229, 250)	226 (217, 236)	0.71
Sweets/desserts	246 (235, 258)	238 (228, 247)	231 (222, 240)	225 (216, 234)	224 (215, 233)	0.002
Olive oil	238(225, 252)	235(225, 245)	236(228, 245)	231(205, 261)	226(220, 233)	0.03

FlOP, fluorescent oxidation product. * Adjusted for variables used in Table 2; ^†^ *p*-trend was calculated by modeling the median value within the categories of dietary intake as a continuous variable and calculating the Wald test statistic; ^‡^ The median intakes of individual food groups within the categories of increasing quintile are as follows: 1.54, 2.42, 3.23, 4.15 and 5.9 servings/day for total vegetables; 0, 0.14, 0.50, 1.00 and 1.28 servings/day for yellow/orange vegetables; 0.07, 0.14, 0.28, 0.57 and 1.00 servings/day for leafy vegetables; 0.49, 1.06, 1.50, 2.06 and 3.14 servings/day for cruciferous vegetables; 0.14, 0.43, 0.64, 0.93 and 1.50 servings/day for other vegetables; 0.49, 1.06, 1.50, 2.06 and 3.14 servings/day for total fruits; 0.42, 0.78, 1.21, 1.71 and 3.14 servings/day for nuts; 0.14, 0.21, 0.50, 0.57 and 1.00 servings/day for legumes; 0.07, 0.14, 0.21, 0.43 and 0.50 servings/day for whole grains; 0.21, 0.35, 0.56, 0.85 and 1.42 servings/day for red/processed meat; 0.07, 0.14, 0.21, 0.35, and 0.64 servings/day for poultry; 0.07, 0.28, 0.35, 0.56 and one serving/day for fish; 0, 1.80, 9.90, 19.50 and 36.00 g/day for alcohol consumption; 0.21, 0.35, 0.56, 0.85 and 1.42 servings/day for sugar-sweetened beverages; and 0.21, 0.56, 0.99, 1.51 and 2.85 servings/day for sweets/desserts.

## Supplementary Materials

The following are available online at http://www.mdpi.com/2072-6643/8/9/587/s1, Figure S1. A diagram for the development of the study sample. Table S1. Individual food components of healthy eating pattern scores. Table S2. Significant interaction observed between healthy dietary scores and fluorescent oxidation product (FlOP) measurements by smoking status and body mass index. Table S3. Multivariate-adjusted * geometric mean concentrations of FlOP_320 and FlOP_400 (FI/mL) by quintile (Q) of food group or individual food intake from the Nurses’ Health Study. Table S4. Multivariate-adjusted * geometric mean concentrations of FlOP_360, FlOP_320 and FlOP400 (FI/mL) by quintile (Q) of individual nutrient intakes from the Nurses’ Health Study.

## Abbreviations

AHEI	Alternative Healthy Eating Index
aMED	the Alternate Mediterranean Diet
BMI	body mass index
CI	confidence interval
CV	coefficients of variation
DASH	the Dietary Approach to Stop Hypertension
FFQ	food frequency questionnaire
FlOP	fluorescent oxidation products
FI	fluorescent intensity units
MDA	malondialdehyde
NHS	Nurses’ Health Study
