# Supplementary material for: Healthy Dietary Patterns and Oxidative Stress as Measured by Fluorescent Oxidation Products in Nurses’ Health Study
Source: Nutrients. 2016 Sep 21;8(9):587. doi: 10.3390/nu8090587 (PMC5037570; doi:10.3390/nu8090587)
Supplement: Supplementary file 1 [file nutrients-08-00587-s001.docx]

Supplementary Materials: Healthy Dietary Patterns and Oxidative Stress as Measured by Fluorescent Oxidation Products in Nurses’ Health Study

Seungyoun Jung, Stephanie A. Smith–Warner, Walter C. Willett, Molin Wang, Tianying Wu, Majken Jensen, Susan E. Hankinson and A. Heather Eliassen

**Table S1.** Individual food components of healthy eating pattern scores.

| **Alternate Healthy Eating Index (9 Components)** | **Alternate Mediterranean Diet  (9 Components)** | **DASH Diet (8 Components)** |
| --- | --- | --- |
| Vegetables | Vegetables | Vegetables |
| Fruit | Fruits | Fruits |
| Nuts and soy | Nuts | Nuts and Legumes |
|  | Legumes |  |
| Cereal fiber | Whole grains | Whole grains |
| Ratio of white to red meat | Red/processed meats | Red/processed meats |
| Transfat (% energy) |  |  |
|  |  | Low–fat dairy |
| Polyunsaturated: saturated fat ratio | Monounsaturated: saturated fat ratio |  |
|  | Fish |  |
| Alcohol | Alcohol |  |
|  |  | Sodium |
|  |  | Sweetened beverages |
| Duration of vitamin use |  |  |

**Table S2.** Significant interaction observed between healthy dietary scores and fluorescent oxidation product (FlOP) measurements by smoking status and body mass index.

| **Food Group/Individual Food ^‡^** | **Quintiles of Dietary Intake** | | | | | ***p*-Trend ^†^** | ***P*-Interaction** **^‡^** |
| --- | --- | --- | --- | --- | --- | --- | --- |
|  | **Q1** | **Q2** | **Q3** | **Q4** | **Q5** |  |  |
| FlOP_320 |  |  |  |  |  |  |  |
| DASH score |  |  |  |  |  |  |  |
| BMI < 25 | 564 (479–664) | 576 (500–664) | 529 (464–604) | 565 (484–660) | 584 (510–669) | 0.76 | 0.01 |
| BMI > 25 | 457 (387–539) | 465 (406–532) | 554 (484–634) | 551 (472–644) | 647 (563–744) | <0.001 |  |
| FlOP_400 |  |  |  |  |  |  |  |
| AHEI score |  |  |  |  |  |  |  |
| Never–smoke | 56 (53–59) | 58 (55–62) | 56 (53–60) | 58 (55–61) | 57 (54–60) | 0.73 | 0.01 |
| Ever–smoke | 71 (67–75) | 68 (64–72) | 70 (67–73) | 72 (69–76) | 69 (65–72) | 0.87 |  |

Abbreviation: FlOP, fluorescent oxidation product. ^†^ *p*-trend was calculated by modeling the median value within the categories of dietary intake as continuous variable and calculating the Wald test statistic. **^‡^** *P*-interaction was calculated by including the cross-product term between stratification factors and the dietary pattern score in the multivariable model.

**Table S3.** Multivariate-adjusted * geometric mean concentrations of FlOP_320 and FlOP_400 (FI/mL) by quintile (Q) of food group or individual food intake from the Nurses’ Health Study.

| **Food Group/Individual Food ^‡^** | **Quintiles of Dietary Intake** | | | | | ***p*-Trend ^†^** |
| --- | --- | --- | --- | --- | --- | --- |
|  | **Q1** | **Q2** | **Q3** | **Q4** | **Q5** |  |
| FlOP_320 |  |  |  |  |  |  |
| Total vegetables | 513 (466–566) | 502 (457–552) | 552 (500–610) | 540 (487–598) | 673 (598–758) | <0.001 |
| Yellow/orange vegetables | 547 (484–618) | 593 (535–657) | 553 (503–608) | 518 (467–574) | 545 (497–598) | 0.26 |
| Leafy vegetables | 539 (477–609) | 530 (478–588) | 553 (509–600) | 554 (501–613) | 582 (523–648) | 0.26 |
| Cruciferous vegetables | 546 (493–604) | 491 (448–538) | 539 (491–592) | 544 (492–601) | 656 (580–743) | 0.007 |
| Other vegetables | 507 (463–556) | 531 (484–584) | 591 (527–663) | 544 (495–598) | 597 (537–664) | 0.05 |
| Total fruits | 546 (493–605) | 491 (449–537) | 540 (492–594) | 545 (493–603) | 653 (577–739) | 0.008 |
| Nut | 627 (559–703) | 554 (503–609) | 561 (509–619) | 551 (497–610) | 489 (442–541) | 0.005 |
| Legume | 554 (488–629) | 529 (484–578) | 509 (461–562) | 604 (546–669) | 565 (513–621) | 0.37 |
| Whole grains | 541 (474–618) | 562 (505–626) | 576 (509–651) | 531 (485–582) | 559 (515–608) | 0.83 |
| Red/processed meat | 521 (469–579) | 558 (497–626) | 539 (494–589) | 575 (522–633) | 578 (517–647) | 0.23 |
| Poultry | 575 (510–649) | 523 (471–582) | 527 (472–588) | 549 (507–594) | 595 (534–664) | 0.20 |
| Fish | 496 (440–559) | 539 (485–598) | 583 (528–645) | 550 (505–600) | 582 (523–647) | 0.17 |
| Alcohol | 550 (508–596) | 537 (499–578) | 581 (522–646) | 523 (442–619) | 635 (511–789) | 0.43 |
| Sugar-sweetened beverages | 521 (469–579) | 558 (497–626) | 539 (494–589) | 574 (522–632) | 579 (517–648) | 0.22 |
| Sweets/desserts | 590 (528–658) | 539 (489–593) | 563 (509–622) | 521 (472–574) | 558 (499–625) | 0.73 |
| Olive oil | 537(472V612) | 556(507–611) | 545(499–594) | 818(493–1359) | 558(519–599) | 0.71 |
| FIOP_400 |  |  |  |  |  |  |
| Total vegetables | 65 (63V68) | 63 (61–66) | 64 (62–66) | 63 (61–65) | 66 (64–69) | 0.42 |
| Yellow/orange vegetables | 64 (61–67) | 64 (62–66) | 65 (63–68) | 65 (62–67) | 64 (62–66) | 0.75 |
| Leafy vegetables | 66 (63–70) | 64 (62–67) | 64 (62–66) | 64 (62–67) | 64 (61–66) | 0.51 |
| Cruciferous vegetables | 64 (62–67) | 65 (62–68) | 66 (63–68) | 63 (61–65) | 64 (61–66) | 0.46 |
| Other vegetables | 65 (62–67) | 65 (62–67) | 63 (61–65) | 63 (61–65) | 66 (63–69) | 0.56 |
| Total fruits | 64 (62–67) | 65 (62–68) | 66 (63–68) | 63 (61–65) | 64 (61–66) | 0.46 |
| Nuts | 64 (62–67) | 63 (61–66) | 66 (63–68) | 65 (63–67) | 63 (61–66) | 0.71 |
| Legume | 63 (60–66) | 63 (61–65) | 64 (62–67) | 65 (63–68) | 66 (63–68) | 0.05 |
| Whole grains | 64 (61–68) | 63 (61–66) | 65 (62–68) | 64 (62–66) | 65 (63–67) | 0.75 |
| Red/processed meat | 63 (60–65) | 65 (62–68) | 64 (62–66) | 66 (64–69) | 64 (61–66) | 0.58 |
| Poultry | 68 (64–72) | 64 (61–66) | 62 (60–65) | 64 (62–66) | 65 (62–67) | 0.72 |
| Fish | 62 (60–65) | 66 (63–69) | 63 (61–65) | 66 (64–69) | 63 (61–66) | 0.94 |
| Alcohol | 63 (61–65) | 64 (62–65) | 67 (64–70) | 64 (61–68) | 69 (63–74) | 0.02 |
| Sugar–sweetened beverages | 63 (60–65) | 65 (62–68) | 64 (62–66) | 66 (64–69) | 64 (61–66) | 0.60 |
| Sweets/desserts | 67 (64–69) | 64 (62–67) | 65 (62–67) | 63 (61–66) | 63 (60–65) | 0.04 |
| Olive oil | 63(60–66) | 65(62–67) | 66(64–68) | 63(57–70) | 64(62–65) | 0.49 |

Abbreviation: FlOP, fluorescent oxidation product. * Adjusted for variables noted in Table 2. ^†^ *p*-trend was calculated by modeling the median value within the categories of dietary intake as a continuous variable and calculating the Wald test statistic. ^‡^ The median intakes of individual food groups within the categories of increasing quintile are as follows: 1.54, 2.42, 3.23, 4.15 and 5.9 servings/day for total vegetables; 0, 0.14, 0.50, 1.00 and 1.28 servings/day for yellow/orange vegetables; 0.07, 0.14, 0.28, 0.57 and 1.00 servings/day for leafy vegetables; 0.49, 1.06, 1.50, 2.06 and 3.14 servings/day for cruciferous vegetables; 0.14, 0.43, 0.64, 0.93 and 1.50 servings/day for other vegetables; 0.49, 1.06, 1.50, 2.06 and 3.14 servings/day for total fruits; 0.42, 0.78, 1.21, 1.71 and 3.14 servings/day for nuts; 0.14, 0.21, 0.50, 0.57 and 1.00 servings/day for legumes; 0.07, 0.14, 0.21, 0.43 and 0.50 servings/day for whole grains; 0.21, 0.35, 0.56, 0.85 and 1.42 servings/day for red/processed meat; 0.07, 0.14, 0.21, 0.35, and 0.64 servings/day for poultry; 0.07, 0.28, 0.35, 0.56 and one servings/day for fish; 0, 1.80, 9.90, 19.50 and 36.00 g/day for alcohol consumption; 0.21, 0.35, 0.56, 0.85 and 1.42 servings/day for sugar–sweetened beverages; and 0.21, 0.56, 0.99, 1.51 and 2.85 servings/day for sweets/desserts.

**Table S4.** Multivariate-adjusted * geometric mean concentrations of FlOP_360, FlOP_320 and FlOP400 (FI/mL) by quintile (Q) of individual nutrient intakes from the Nurses’ Health Study.

|  | **Quintiles of Dietary Intake** | | | | | | ***p*-Trend ^†^** |
| --- | --- | --- | --- | --- | --- | --- | --- |
|  | **Q1** | **Q2** | | **Q3** | **Q4** | **Q5** |  |
| FlOP_360 |  |  | |  |  |  |  |
| Macro-nutrients |  |  | |  |  |  |  |
| Total fat | 235 (226, 246) | 239 (229, 250) | | 231 (222, 241) | 230 (221, 239) | 226 (217, 235) | 0.09 |
| Animal fat | 235 (224, 246) | 233 (224, 243) | | 235 (225, 245) | 229 (220, 239) | 229 (220, 238) | 0.31 |
| Vegetable fat | 239 (229, 249) | 235 (226, 245) | | 228 (219, 238) | 230 (220, 240) | 230 (221, 238) | 0.13 |
| Saturated fat | 241 (230, 252) | 233 (224, 243) | | 239 (229, 250) | 227 (218, 236) | 222 (214, 231) | 0.007 |
| Monounsaturated fat | 236 (226, 246) | 237 (227, 247) | | 230 (222, 239) | 226 (218, 236) | 232 (223, 242) | 0.32 |
| Polyunsaturated fat | 235 (226, 245) | 234 (224, 243) | | 229 (220, 238) | 233 (223, 243) | 231 (222, 240) | 0.53 |
| Trans fat | 246 (236, 257) | 231 (222, 240) | | 233 (223, 243) | 231 (223, 240) | 221 (212, 231) | 0.003 |
| Omega-3 fat | 235 (223, 246) | 228 (219, 238) | | 230 (222, 238) | 235 (225, 244) | 234 (225, 243) | 0.63 |
| Cholesterol | 229 (220, 239) | 236 (226, 246) | | 229 (220, 239) | 235 (226, 245) | 232 (223, 242) | 0.73 |
| Total protein | 229 (220, 238) | 219 (211, 227) | | 235 (224, 246) | 234 (225, 243) | 246 (236, 257) | 0.002 |
| Animal protein | 231 (222, 241) | 223 (215, 232) | | 233 (223, 243) | 231 (222, 241) | 243 (233, 253) | 0.04 |
| Vegetable protein | 221 (213, 230) | 240 (229, 251) | | 227 (219, 235) | 237 (227, 247) | 237 (227, 248) | 0.06 |
| Total carbohydrate | 233 (223, 244) | 232 (223, 241) | | 232 (222, 242) | 234 (224, 243) | 231 (222, 241) | 0.86 |
| Fructose | 227 (217, 237) | 238 (229, 247) | | 232 (222, 242) | 237 (226, 248) | 229 (220, 238) | 0.98 |
| Antioxidants |  |  | |  |  |  |  |
| Vitamin E | 236 (226, 246) | 223 (214, 233) | | 229 (220, 238) | 236 (226, 245) | 239 (229, 249) | 0.15 |
| Vitamin A | 227 (218, 236) | 231 (221, 241) | | 232 (223, 242) | 234 (224, 244) | 237 (227, 247) | 0.16 |
| Vitamin C | 226 (217, 236) | 230 (220, 240) | | 226 (217, 235) | 235 (225, 244) | 245 (235, 256) | 0.003 |
| α-carotene | 233 (223, 243) | 228 (219, 238) | | 233 (224, 243) | 230 (222, 239) | 237 (227, 249) | 0.38 |
| β-carotene | 226 (216, 235) | 230 (221, 239) | | 237 (227, 247) | 229 (220, 238) | 241 (231, 251) | 0.07 |
| β-cryptoxanthin | 232 (222, 242) | 230 (222, 238) | | 224 (215, 232) | 242 (231, 253) | 235 (225, 245) | 0.26 |
| utein/zeaxanthin | 226 (217, 235) | 227 (219, 235) | | 235 (225, 245) | 235 (225, 246) | 239 (229, 249) | 0.04 |
| Lycopene | 221 (213, 229) | 224 (216, 234) | | 231 (222, 240) | 237 (229, 247) | 249 (237, 261) | < 0.001 |
| Total carotenoid | 229 (219, 239) | 230 (222, 239) | | 233 (224, 243) | 234 (225, 244) | 235 (225, 246) | 0.33 |
| Total flavonoid | 228 (218, 238) | 231 (222, 241) | | 231 (223, 239) | 242 (232, 253) | 230 (221, 240) | 0.64 |
| Other nutrients |  |  | |  |  |  |  |
| Fiber | 224 (215, 234) | 225 (217, 234) | | 236 (226, 245) | 230 (222, 239) | 248 (236, 260) | 0.003 |
| Sodium | 241 (231, 252) | 223 (215, 231) | | 238 (228, 249) | 224 (215, 233) | 235 (226, 245) | 0.57 |
| FlOP_320 |  |  | |  |  |  |  |
| Macro-nutrients |  |  | |  |  |  |  |
| Total fat | 532 (481, 588) | 591 (529, 660) | | 562 (508, 621) | 562 (509, 622) | 523 (475, 575) | 0.64 |
| Animal fat | 537 (485, 594) | 543 (493, 598) | | 576 (517, 641) | 556 (501, 616) | 557 (504, 615) | 0.60 |
| Vegetable fat | 583 (523, 649) | 536 (487, 589) | | 558 (505, 617) | 521 (475, 571) | 572 (513, 636) | 0.80 |
| Saturated fat | 535 (482, 594) | 579 (521, 643) | | 607 (544, 677) | 529 (483, 581) | 523 (476, 575) | 0.41 |
| Monounsaturated fat | 552 (497, 614) | 571 (515, 633) | | 595 (537, 659) | 521 (474, 574) | 532 (483, 586) | 0.36 |
| Polyunsaturated fat | 558 (502, 620) | 522 (479, 569) | | 576 (518, 640) | 539 (489, 595) | 574 (518, 635) | 0.58 |
| Trans fat | 592 (531, 661) | 559 (507, 616) | | 537 (488, 591) | 553 (501, 610) | 530 (481, 583) | 0.18 |
| Omega-3 fat | 532 (479, 592) | 540 (488, 596) | | 571 (516, 631) | 543 (493, 599) | 578 (522, 640) | 0.31 |
| Cholesterol | 508 (462, 559) | 573 (516, 635) | | 569 (513, 632) | 542 (492, 597) | 578 (522, 641) | 0.22 |
| Protein | 517 (469, 570) | 501 (456, 551) | | 552 (500, 609) | 604 (543, 671) | 600 (538, 670) | 0.007 |
| Animal protein | 508 (463, 557) | 538 (485, 597) | | 526 (480, 577) | 582 (527, 643) | 620 (554, 693) | 0.005 |
| Vegetable protein | 537 (487, 592) | 541 (492, 595) | | 577 (519, 641) | 607 (543, 678) | 511 (466, 560) | 0.76 |
| Total carbohydrate | 554 (497, 616) | 574 (517, 638) | | 545 (493, 604) | 576 (519, 640) | 521 (473, 573) | 0.44 |
| Fructose | 520 (472, 573) | 584 (526, 647) | | 552 (500, 610) | 565 (512, 624) | 548 (493, 609) | 0.75 |
| Antioxidants |  |  | |  |  |  |  |
| Vitamin E | 486 (443, 534) | 511 (462, 565) | | 498 (450, 550) | 599 (542, 662) | 699 (627, 779) | <0.001 |
| Vitamin A | 470 (429, 514) | 496 (454, 542) | | 600 (538, 668) | 581 (523, 646) | 638 (574, 708) | <0.001 |
| Vitamin C | 496 (450, 548) | 497 (453, 546) | | 494 (450, 542) | 609 (549, 676) | 697 (625, 778) | <0.001 |
| α, carotene | 535 (484, 590) | 520 (475, 570) | | 566 (513, 623) | 546 (490, 609) | 604 (542, 672) | 0.06 |
| β, carotene | 498 (453, 548) | 522 (477, 570) | | 584 (525, 650) | 531 (480, 587) | 641 (576, 714) | 0.001 |
| β, cryptoxanthin | 541 (492, 594) | 561 (505, 623) | | 545 (493, 603) | 565 (509, 626) | 556 (502, 616) | 0.73 |
| Lutein/zeaxanthin | 500 (456, 549) | 564 (510, 625) | | 534 (485, 587) | 580 (520, 647) | 593 (535, 658) | 0.03 |
| Lycopene | 560 (506, 619) | 491 (449, 537) | | 594 (536, 657) | 546 (492, 605) | 584 (528, 646) | 0.23 |
| Total carotenoid | 509 (464, 559) | 505 (461, 552) | | 592 (533, 657) | 574 (516, 639) | 594 (533, 661) | 0.02 |
| Total flavonoid | 500 (458, 546) | 560 (505, 620) | | 577 (518, 643) | 558 (502, 619) | 576 (521, 636) | 0.15 |
| Other nutrients |  |  | |  |  |  |  |
| Fiber | 500 (457, 548) | 517 (470, 569) | | 581 (525, 643) | 549 (496, 609) | 626 (558, 703) | 0.004 |
| odium | 610 (549, 677) | 517 (472, 567) | | 562 (505, 626) | 545 (492, 602) | 537 (488, 592) | 0.20 |
| FlOP_400 |  |  | |  |  |  |  |
| Macro-nutrients |  |  | |  |  |  |  |
| Total fat | 64 (62, 67) | 64 (61, 66) | | 65 (62, 67) | 64 (62, 67) | 64 (62, 67) | 0.72 |
| Animal fat | 65 (62, 68) | 63 (61, 66) | | 65 (62, 67) | 63 (61, 66) | 65 (63, 67) | 0.90 |
| Vegetable fat | 64 (62, 67) | 65 (63, 68) | | 63 (61, 66) | 65 (62, 67) | 64 (61, 66) | 0.55 |
| Saturated fat | 65 (62, 68) | 63 (61, 65) | | 66 (63, 68) | 63 (61, 66) | 64 (62, 67) | 0.72 |
| Monounsaturated fat | 64 (62, 67) | 64 (62, 66) | | 64 (62, 67) | 65 (62, 67) | 65 (62, 67) | 0.65 |
| Polyunsaturated fat | 64 (62, 67) | 65 (62, 67) | | 63 (61, 65) | 65 (62, 67) | 64 (62, 67) | 0.99 |
| Trans fat | 65 (62, 67) | 63 (61, 65) | | 65 (63, 68) | 65 (63, 68) | 63 (60, 65) | 0.49 |
| Omega-3 fat | 66 (64, 70) | 64 (61, 66) | | 63 (61, 65) | 66 (63, 68) | 63 (61, 65) | 0.28 |
| Cholesterol | 64 (61, 67) | 64 (62, 67) | | 63 (61, 65) | 66 (63, 68) | 65 (62, 67) | 0.51 |
| Protein | 65 (63, 68) | | 61 (59, 63) | 66 (63, 69) | 64 (62, 66) | 65 (63, 68) | 0.45 |
| Animal protein | 66 (63, 68) | | 62 (60, 64) | 66 (63, 68) | 63 (61, 66) | 65 (63, 67) | 0.90 |
| Vegetable protein | 63 (61, 66) | | 66 (63, 69) | 62 (60, 64) | 64 (62, 67) | 65 (63, 68) | 0.50 |
| Total carbohydrate | 66 (63, 69) | | 64 (61, 66) | 65 (62, 68) | 64 (62, 66) | 63 (61, 65) | 0.16 |
| Fructose | 64 (62, 67) | | 66 (64, 69) | 64 (62, 67) | 65 (62, 68) | 62 (60, 64) | 0.06 |
| Antioxidants |  | |  |  |  |  |  |
| Vitamin E | 66 (64, 69) | | 64 (61, 66) | 62 (60, 64) | 64 (62, 67) | 65 (62, 67) | 0.63 |
| Vitamin A | 65 (63, 68) | | 65 (63, 68) | 62 (60, 65) | 64 (62, 67) | 64 (62, 67) | 0.40 |
| Vitamin C | 65 (63, 67) | | 66 (63, 68) | 63 (61, 65) | 62 (60, 64) | 66 (63, 68) | 0.44 |
| α, carotene | 64 (62, 67) | | 64 (62, 66) | 65 (63, 68) | 63 (61, 66) | 65 (62, 68) | 0.80 |
| β, carotene | 64 (62, 67) | | 65 (63, 68) | 64 (62, 66) | 63 (61, 66) | 65 (62, 67) | 0.96 |
| β, cryptoxanthin | 65 (62, 68) | | 63 (61, 65) | 64 (62, 67) | 66 (64, 69) | 63 (61, 65) | 0.71 |
| Lutein | 64 (62, 66) | | 64 (62, 66) | 65 (62, 67) | 64 (62, 67) | 64 (62, 67) | 0.82 |
| Lycopene | 63 (61, 65) | | 63 (60, 65) | 65 (63, 68) | 65 (62, 67) | 66 (63, 69) | 0.08 |
| Total carotenoid | 64 (62, 67) | | 64 (62, 66) | 65 (63, 68) | 64 (62, 67) | 64 (61, 66) | 0.70 |
| Total flavonoid | 67 (65, 70) | | 64 (62, 66) | 64 (62, 67) | 64 (62, 67) | 61 (59, 64) | 0.006 |
| Other nutrients |  | |  |  |  |  |  |
| Fiber | 65 (63, 68) | | 63 (61, 65) | 66 (64, 69) | 63 (61, 65) | 64 (62, 67) | 0.80 |
| Sodium | 66 (63, 68) | | 63 (61, 65) | 65 (62, 67) | 62 (60, 64) | 66 (64, 69) | 0.58 |

Abbreviation: FlOP, fluorescent oxidation product. * Adjusted for variables used in Table 2. ^†^ *p*, trend was calculated by modeling the median value within the categories of dietary intake as continuous variable and calculating the Wald test statistic.


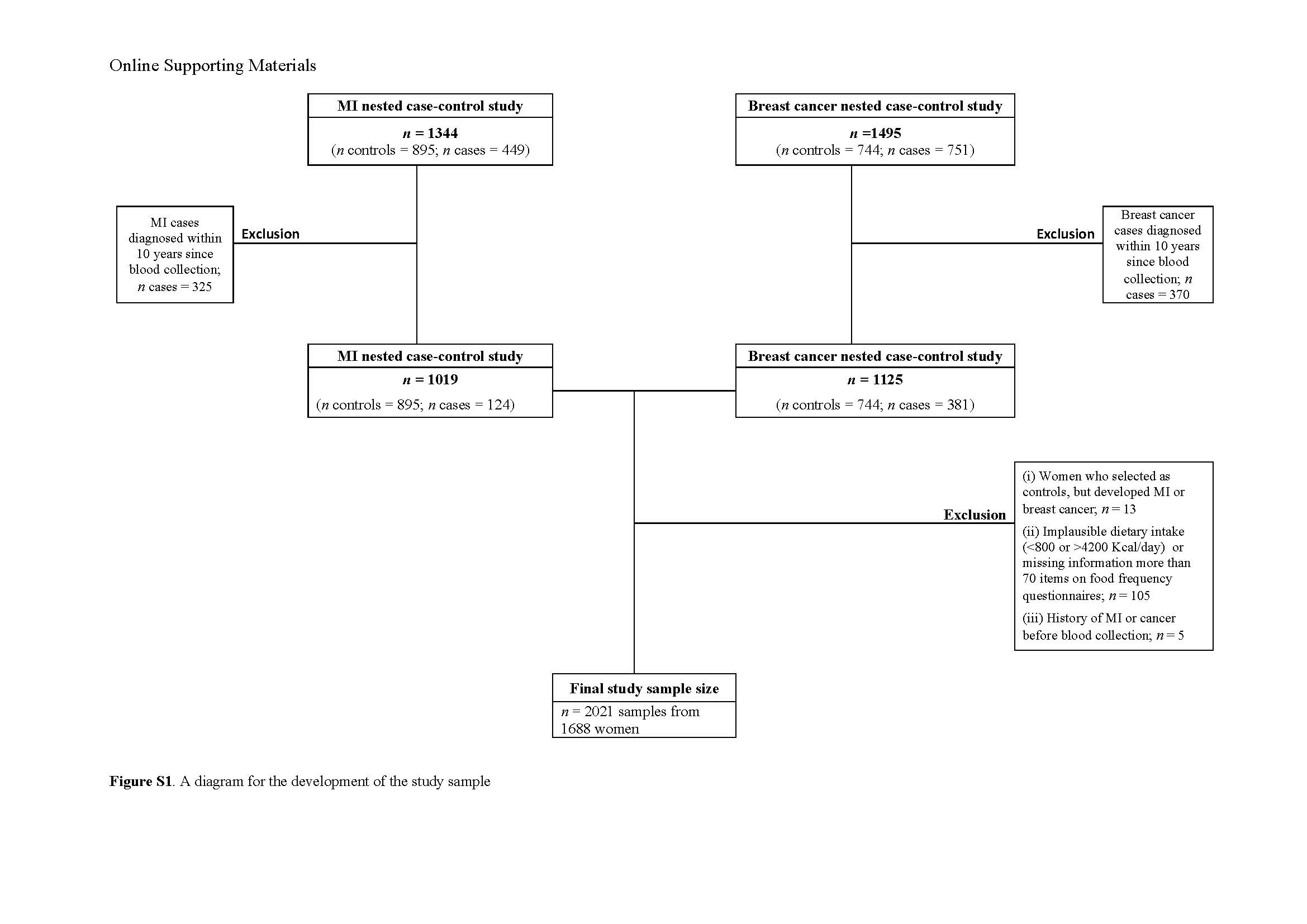


**Figure S1.** A diagram for the development of the study sample.
